# Supplementary material for: Mouse models of tuberculosis uniformly featuring hypoxic necrotic lesions and poor response to chemotherapy
Source: Infect Immun. 2026 Feb 9;94(3):e00574-25. doi: 10.1128/iai.00574-25 (PMC12974119; doi:10.1128/iai.00574-25)
Supplement: Supplemental material — Supplemental methods; Fig. S1 to S5, Tables S1 and S2. [file iai.00574-25-s0001.pdf]

Supplementary Methods

Hypoxic Lesion Analysis: All images were edited to ensure a uniform white background and analyzed using FIJI/ImageJ. Total image area was measured directly from the resulting image. Total tissue area was then quantified by converting each image to binary and using the measure function to calculate the area of the lung tissue. Stain separation was performed using the Colour Deconvolution plugin with custom ROI-derived vectors, producing individual Hematoxylin, Vector Red (VR), and background channels. The background channel was discarded for this analysis. Hematoxylin-positive area was quantified by converting each Hematoxylin image to binary and measuring the area with the measure function. VR quantification was calibrated using a negative control slide lacking pimonidazole: brightness/contrast (B/C) settings, specifically the Max value, were decreased until binary conversion resulted in zero measurable VR-positive area, ensuring the elimination of false-positive background signal. These calibrated B/C settings were then applied identically to all experimental VR channel images, which were subsequently converted to binary and measured to obtain VR-positive area.

Necrotic Lesion Analysis: All images were edited to ensure a uniform white background and analyzed using FIJI/ImageJ. Total tissue area was then quantified by converting each image to binary and using the measure function to calculate the area of the lung tissue. The lesions were identified by eye to assess the presence of necrosis (non-nucleated areas within granulomas, well-formed macrophages layers surrounding lesions, etc.). These lesions were then outlined using the free-hand drawing tool along the boundary just inside the macrophage layer. The measure function was then used to calculate the area of each lesion.

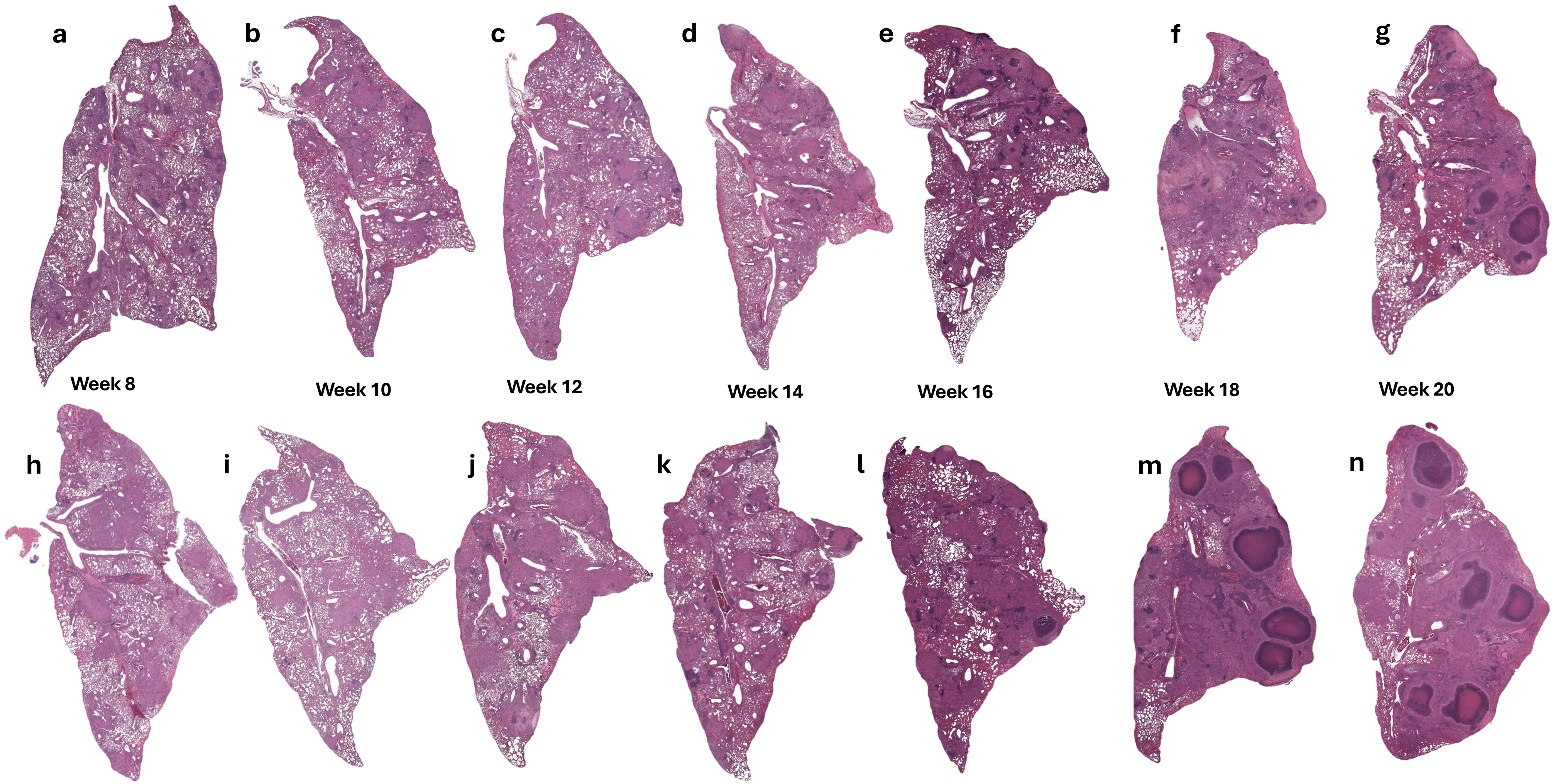
a
b
c
d
e
f
g
Week 8
Week 10
Week 12
Week 14
Week 16
Week 18
Week 20
h
i
j
k
l
m
n

Supplementary Figure 1. Histopathological progression of lung lesions in *Nos2*$^{-/-}$ mice during the naïve model of *Mtb* infection. Hematoxylin and eosin (H&E) staining of representative left lung sections from *Nos2*$^{-/-}$ mice infected with *Mtb* R1Rv. Each panel depicts lesion progression at two-week intervals, beginning at week 8 and continuing through week 20 post-infection. Top row (a–g) shows female mice; bottom row (h–n) shows male mice. (Magnification 10×)

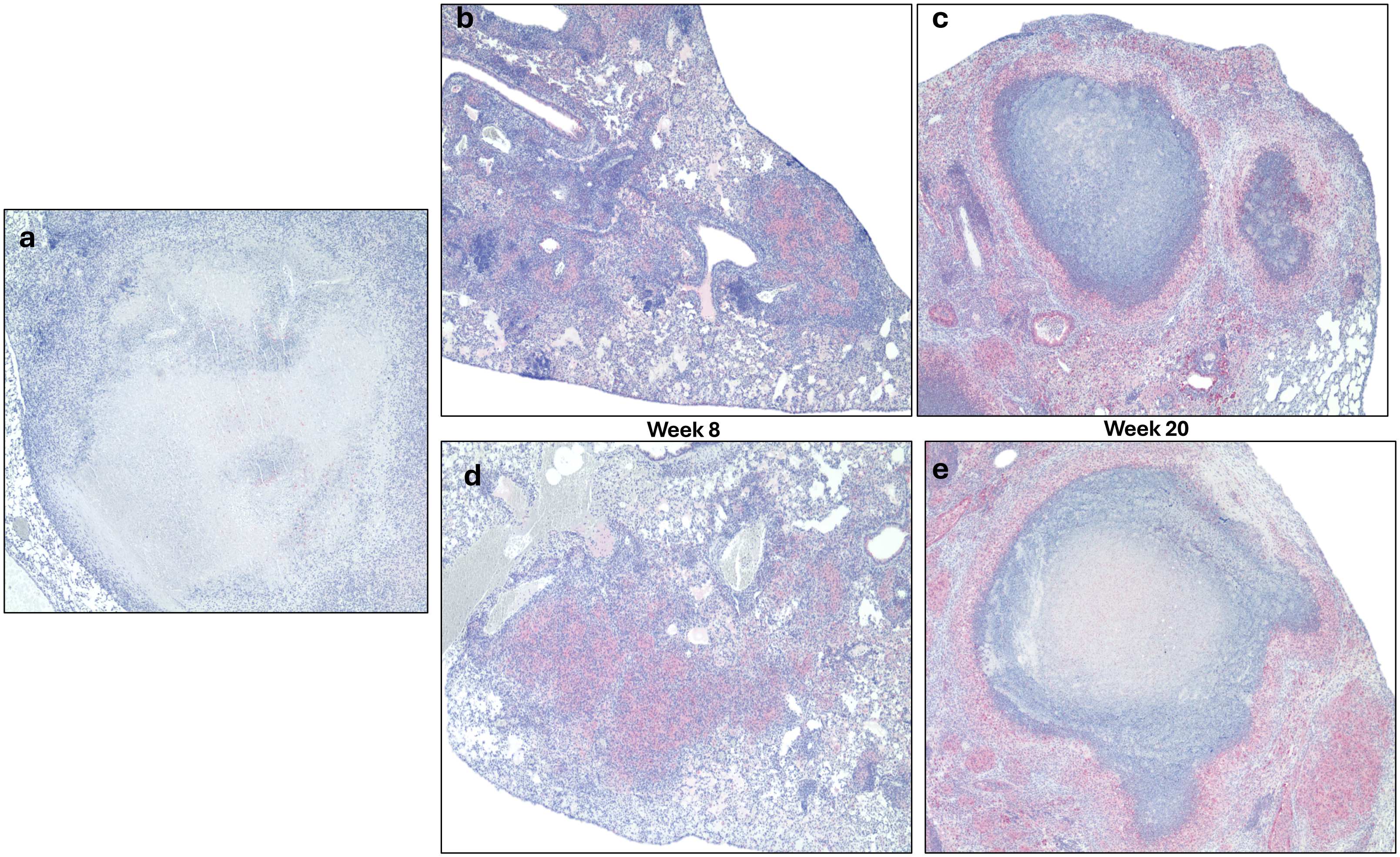
a
b
c
Week 8
Week 20
d
e

Supplementary Figure 2: **Detection of hypoxia in developing necrotic lung lesions of *Nos2*$^{-/-}$ mice in the naïve model.**

Pimonidazole staining was used to detect hypoxic regions within lung lesions of naïve *Nos2*$^{-/-}$ mice infected with *Mtb* R1Rv. A week 20 infected mouse that did not receive pimonidazole served as a negative control to confirm staining specificity (a). At week 8 post-infection, pimonidazole adducts (pink staining) were diffusely distributed within the lesions, whereas by week 20, staining formed a distinct hypoxic rim surrounding the necrotic cores. Panels b and c show lung sections from female mice at weeks 8 and 20, respectively; panels d and e show male mice at the same time points. (Magnification: 40×)

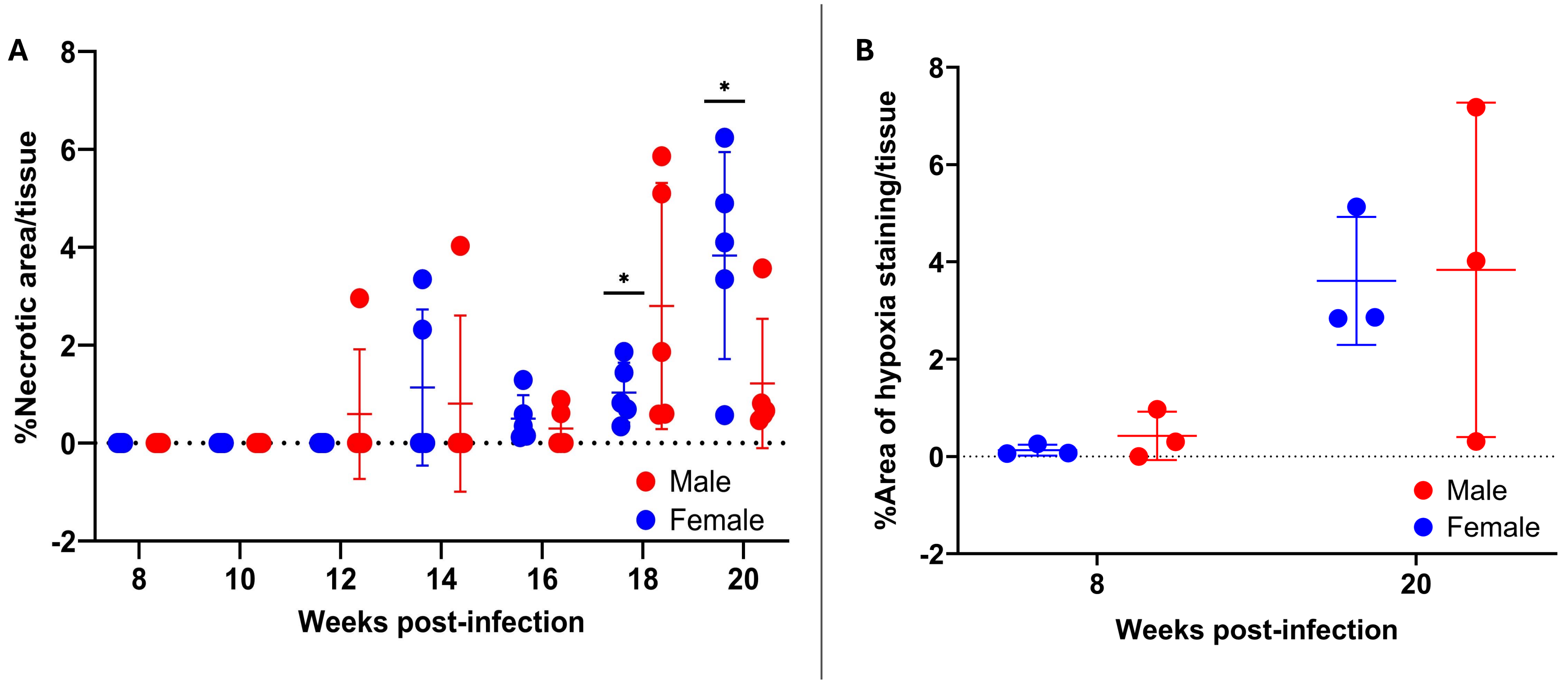
A
%Necrotic area/tissue
8
6
4
2
0
-2
8
10
12
14
16
18
20
Weeks post-infection
*
*
Male
Female
B
%Area of hypoxia staining/tissue
8
6
4
2
0
-2
8
20
Weeks post-infection
Male
Female

Supplementary Figure 3. Percentage of necrotic regions and area of hypoxic staining across tissues of *Nos2*$^{-/-}$ mice in the naïve model

Panel A: Necrotic area was quantified by delineating the necrotic region in each tissue section and normalizing it to the total tissue area. Each data point represents an individual mouse (n = 5).

Panel B: Quantification of hypoxic area was performed using pimonidazole-stained lung sections, with each data point corresponding to an individual mouse (n = 3).

Female data points are shown in blue, and male data points in red. Values are reported as mean ± s.d

No significant differences were observed at any time point in the male groups for either the necrotic regions or the hypoxic staining across tissues. However, in the female group, a significant difference in necrotic regions was detected at weeks 18 and 20 ($p < 0.05$), while all other measures remained non-significant.

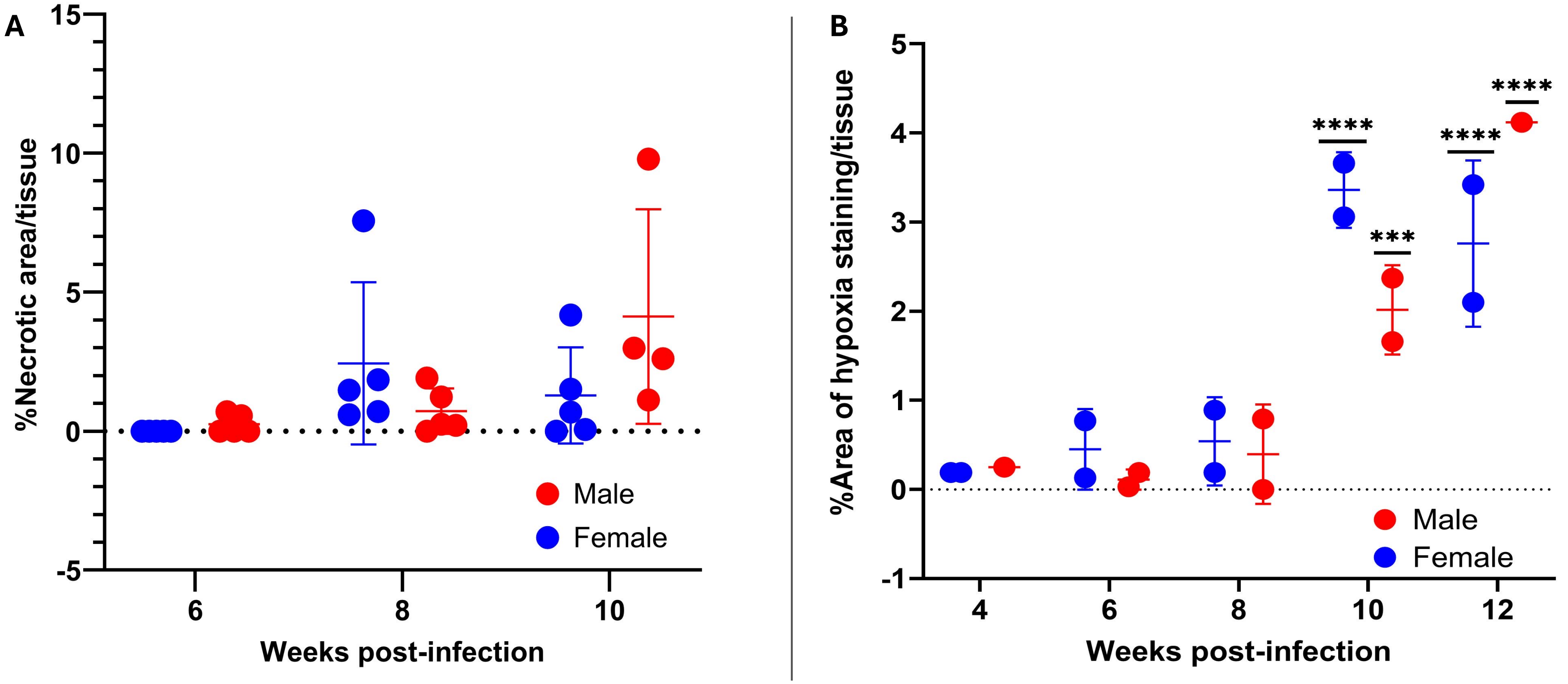
A
15
10
5
0
-5
%Necrotic area/tissue
6
8
10
Weeks post-infection
Male
Female
B
5
4
3
2
1
0
-1
%Area of hypoxia staining/tissue
****
***
****
****
4
6
8
10
12
Weeks post-infection
Male
Female

Supplementary Figure 4. Percentage of necrotic regions and area of hypoxic staining across tissues in vaccinated *Nos2*$^{-/-}$ mice infected with *Mtb* Erdman

Panel A: Necrotic area was quantified by delineating the necrotic region in each tissue section and normalizing it to the total tissue area. Each data point represents an individual mouse (n = 5).

Panel B: Quantification of hypoxic area was performed using pimonidazole-stained lung sections, with each data point corresponding to an individual mouse (n = 2).

Female data points are shown in blue, and male data points in red. Values are reported as mean ± s.d

No significant differences were observed at any time point in the male groups for necrotic regions across tissues. However, in both the male and female groups, a significant difference in hypoxic staining across tissues was detected at weeks 10 and 12 ($p < 0.05$), while all other time points remained non-significant.

For clarity of presentation, one male at week 10 was excluded because of extreme levels of necrosis and one male at weeks 12 was excluded due to extreme hypoxia staining.

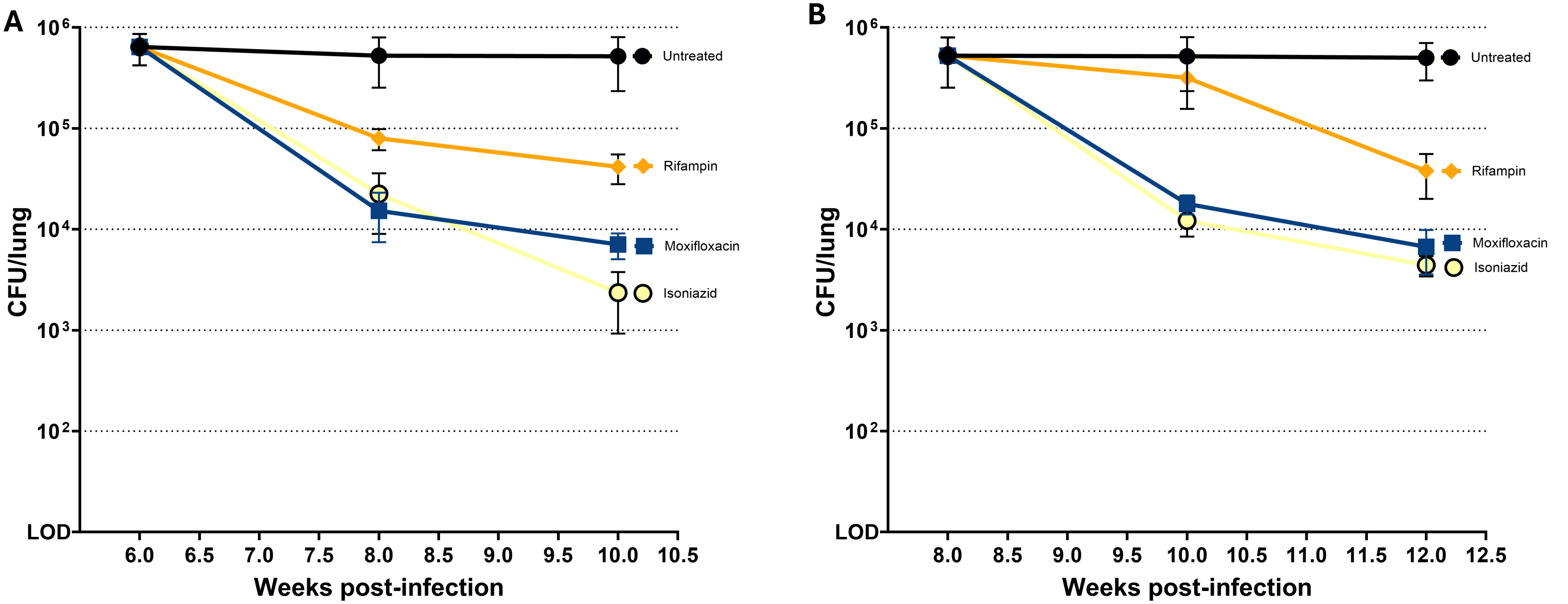
A
B
CFU/lung
10^6
10^5
10^4
10^3
10^2
LOD
6.0 6.5 7.0 7.5 8.0 8.5 9.0 9.5 10.0 10.5
8.0 8.5 9.0 9.5 10.0 10.5 11.0 11.5 12.0 12.5
Weeks post-infection
Untreated
Rifampin
Moxifloxacin
Isoniazid

Supplementary Figure 5. Drug Efficacy in naïve *Nos2*$^{-/-}$ Mice Infected with *Mtb R1Rv* at 6 and 8-weeks post-infection.

Drug treatment was initiated either 6 or 8-weeks post-infection and continued for 4 weeks. Drugs were administered by oral gavage 5 days per week using the following doses: isoniazid, 25 mg/kg (yellow); moxifloxacin, 200 mg/kg (blue); and rifampicin, 10 mg/kg (orange). Each data point represents the mean ± s.e.m. from 5 female mice per group.

Supplementary Table 1: Drug efficacy in vaccinated *Nos2*$^{-/-}$ Mice Infected with *Mtb* Erdman.

Log_{10} CFU changes were calculated relative to the week 6 baseline after 2 and 4 weeks of treatment (weeks 8 and 10, respectively), or relative to the week 8 baseline after 2 weeks of treatment (week 10). Positive values indicate an increase in bacterial burden, whereas negative values indicate a reduction. Statistical analyses were performed relative to the baseline using two-way ANOVA followed by Tukey's multiple comparisons test, with significance determined using adjusted *P* values. Data are presented as mean ± s.e.m. (n = 8–10 per group, both sexes).

**week 6 post-infection baseline**				
**2-week treatment (week 8)**	Dose mg/kg	log_{10} CFU	Adjusted P Value	Signifi-cance
Untreated		0.56		
Pyrazinamide	150	0.37	0.1198	ns
Linezolid	100	0.19	0.3944	ns
Pretomanid	75	-0.19	0.6726	ns
Rifampin	10	-0.76	0.0059	**
Isoniazid	25	-0.72	0.0072	**
Bedaquiline	25	-0.72	0.0066	**
Moxifloxacin	200	-1.64	0.0019	**
**4-week treatment (week 10)**				
Untreated		0.85		
Pyrazinamide	150	0.23	0.4238	ns
Linezolid	100	-0.23	0.7417	ns
Pretomanid	75	-0.85	0.0047	**
Rifampin	10	-1.4	0.0022	**
Isoniazid	25	-1.59	0.002	**
Bedaquiline	25	-1.99	0.0018	**
Moxifloxacin	200	-3.78	0.0017	**
**week 8 post-infection baseline**				
**2-week treatment (week 10)**	Dose mg/kg	log_{10} CFU	Adjusted P Value	Signifi-cance
Untreated		0.87		
Pyrazinamide	150	0.44	0.1557	ns
Linezolid	100	0.27	0.5083	ns
Pretomanid	75	0.05	>0.9999	ns
Rifampin	10	0.35	0.6881	ns
Isoniazid	25	0.08	>0.9999	ns
Bedaquiline	25	-0.39	0.0747	ns
Moxifloxacin	200	-1.66	0.0031	**

Supplementary Table 2: **Drug efficacy in naïve *Nos2*$^{-/-}$ Mice Infected with *Mtb R1Rv*.**

Log_{10} CFU changes were calculated relative to the week 6 baseline after 2 and 4 weeks of treatment (weeks 8 and 10), or relative to the week 8 baseline after 2 and 4 weeks of treatment (weeks 10 and 12). Positive values indicate increased bacterial burden, whereas negative values indicate reduction. Statistical comparisons to baseline were performed using two-way ANOVA with Tukey's multiple comparisons test (adjusted *P* values). Data are shown as mean ± s.e.m. (n = 5 per group, female).

**week 6 post-infection baseline**				
**2-week treatment (week 8)**	Dose mg/kg	$\log_{10}$ CFU reductions	Adjusted P Value	Signifi-cance
Untreated		-0.09		
Rifampin	10	-0.91	0.0169	*
Isoniazid	25	-1.46	0.0069	**
Moxifloxacin	200	-1.62	0.0062	**
**4-week treatment (week 10)**				
Untreated		-0.09		
Rifampin	10	-1.19	0.0094	**
Isoniazid	25	-2.44	0.005	**
Moxifloxacin	200	-1.96	0.0054	**
**week 8 post-infection baseline**				
**2-week treatment (week 10)**	Dose mg/kg	$\log_{10}$ CFU reductions	Adjusted P Value	Signifi-cance
Untreated		0.01		
Rifampin	10	-0.22	0.6722	ns
Isoniazid	25	-1.63	0.0347	*
Moxifloxacin	200	-1.47	0.0375	*
**4-week treatment (week 12)**	Dose mg/kg	$\log_{10}$ CFU reductions	Adjusted P Value	Signifi-cance
Untreated		-0.02		
Rifampin	10	-1.14	0.0492	*
Isoniazid	25	-2.07	0.0311	*
Moxifloxacin	200	-1.89	0.0321	*
